# Nanoparticle trends and hotspots in lung cancer diagnosis from 2006-2023: a bibliometric analysis

**DOI:** 10.3389/fonc.2024.1453021

**Published:** 2024-12-20

**Authors:** Wang Yilun, Zhang Yaojing, Shi Hongcan

**Affiliations:** ^1^ Clinical Medical College, Yangzhou University, Yangzhou, Jiangsu, China; ^2^ Institute of Translational Medicine, Medical College, Yangzhou University, Yangzhou, Jiangsu, China; ^3^ Department of Thoracic and Cardiovascular Surgery, Northern Jiangsu Peoples Hospital Affiliated to Yangzhou University, Yangzhou, Jiangsu, China

**Keywords:** lung cancer, nanoparticles, diagnosis, bibliometric analysis, systematic review

## Abstract

**Background:**

Lung cancer possesses the highest incidence and mortality rates among malignancies globally. Despite substantial advancements in oncology, it is frequently diagnosed at an advanced stage, resulting in a poor prognosis. Over recent decades, the swift progress of nanotechnology has precipitated the extensive utilization of nanomaterials as carriers in cancer diagnosis and therapy. The deployment of nanoparticles as an innovative diagnostic strategy aspires to enable the earlier detection of lung cancer, thereby permitting earlier intervention and enhancing prognosis. This study endeavors to deepen our understanding of this domain through a comprehensive analysis employing bibliometric tools.

**Method:**

Related articles were retrieved from the Web of Science Core Collection from January 1st, 2006, to December 14st, 2023. Thereaf CiteSpace, VOSviewer and the online platform of bibliometrics (http://bibliometric.com/) were utilized to visually analyze Author/Country/Institutions/Cited Journals/Keyword, et al.

**Results:**

A total of 966 articles were retrieved for this study. The analysis unveils a progressive increase in annual publications within this field, with China at the forefront in publication volume, followed by the United States and India. Moreover, Chinese research institutions, notably the Chinese Academy of Sciences and Shanghai Jiao Tong University, prevail in publication output. Upon exclusion of irrelevant search terms, keywords clustering analysis highlights that “biomarkers”, “sensors”, “gold nanoparticles”, and “silver nanoparticles” are predominant research focuses.

**Conclusion:**

This bibliometric study furnishes a quantitative perspective on the extant literature, serving scholars in related fields. Furthermore, it anticipates future research trend concerning nanoparticles and lung cancer diagnosis, thereby aiding in the formulation of project planning and the design of experiments.

## 1 Introduction

Lung cancer is a distinct and heterogeneous disease (1). Recent decades have witnessed a surge in both morbidity and mortality rates associated with lung cancer. It is ranked first in mortality and second in morbidity among all tumors (2). The latest global cancer statistics for 2022 indicate that lung cancer remains the foremost cause of death among cancer patients, accounting for 18.7% (1.817million) of all cancer fatalities (3). The rising number of smokers has precipitated a marked increase in lung cancer incidence (4). Concurrently, the potential risks of lung cancer in non-smokers (comprising 25% of lung cancer patients) cannot be overlooked (5).

Extensive screening and early diagnosis of lung cancer are essential for the prognosis of lung cancer patients. Unfortunately, due to technical bottlenecks, patients with lung cancer often fail to identify the optimal window period for receiving the best treatment, making it a challenging enigma (6, 7). Therefore, procedural screening should be provided as early as possible for patients at high risk of lung cancer to prevent invasive development and systemic metastasis. This strategy can help address the complexities of curing advanced types of lung cancer and significantly improve the patient’s prognosis. Currently, there are six available diagnostic methods for detecting lung cancer: chest radiographs (CXRs) (8), computed tomography (CT) scans (9), magnetic resonance imaging (MRI) (10), positron emission tomography (PET) (11), cytology sputum analysis (12), and breath analysis (13).

Early diagnosis and treatment of lung cancer patients can be significantly advanced through the judicious application of nanotechnology. Nanoparticles (NPs) represent a crucial component of nanotechnology, possessing a unique structure that can amplify the imaging signal of MRI, thereby increasing its detection sensitivity. Moreover, NPs can serve as modification materials for biosensors, enhancing their detection limits and enabling the earlier identification of lung cancer biomarkers. Consequently, this improvement can substantially elevate the rates of early detection (14, 15).

*Bibliometrics* is one of the important methods to objectively measure the impact of academic publications and a discipline dedicated to the statistical and quantitative analysis of published literature, aimed at identifying prevailing issues and scientific trends within a research field (16). And it can elucidate the interconnections among publications and the relationships between authors and countries by sorting and analyzing relevant data such as keywords, references, authorship, and geographical affiliations of the articles (17). The body of research concerning nanotechnology and lung cancer is steadily expanding. However, there exists a paucity of comprehensive and detailed quantitative analyses regarding its current state and developmental trajectories. This study aspires to thoroughly and systematically examine the literature on nanotechnology and lung cancer diagnosis over the past nearly two decades. Additionally, it seeks to forecast future research hotspots, directions, and development trends, thereby providing guidance for researchers in project design and experimental research within this domain.

## 2 Materials and methods

### 2.1 Data sources and search strategies

The precision in document type classification within the Web of Science Core Collection (WoSCC) database surpasses that of alternative databases, thereby establishing it as the preferred option for conducting literature analyses. Consequently, this database was selected for our search. The retrieval and data collection of relevant articles pertaining to the application of nanoparticle in lung cancer were handled and completed on December 14, 2023. The search strategy was revealed as follows: [TS= (lung cancer*) OR TS=(lung tumor*) OR TS=(lung neoplasm*)], [TS=(diagnosis*) OR TS=(Diagnosis and Examinations)], TS=nanoparticle*. Subsequently, we concatenated the above three using the Boolean logic operator “AND” and set the year limit at retrieval to January 1st, 2006, to December 14st, 2023. The document types were Articles and Review Articles. What’s more, the publications were not only unretracted but also not in the “Expression of Concern”.

### 2.2 Inclusion and exclusion criteria

**Figure 1:**
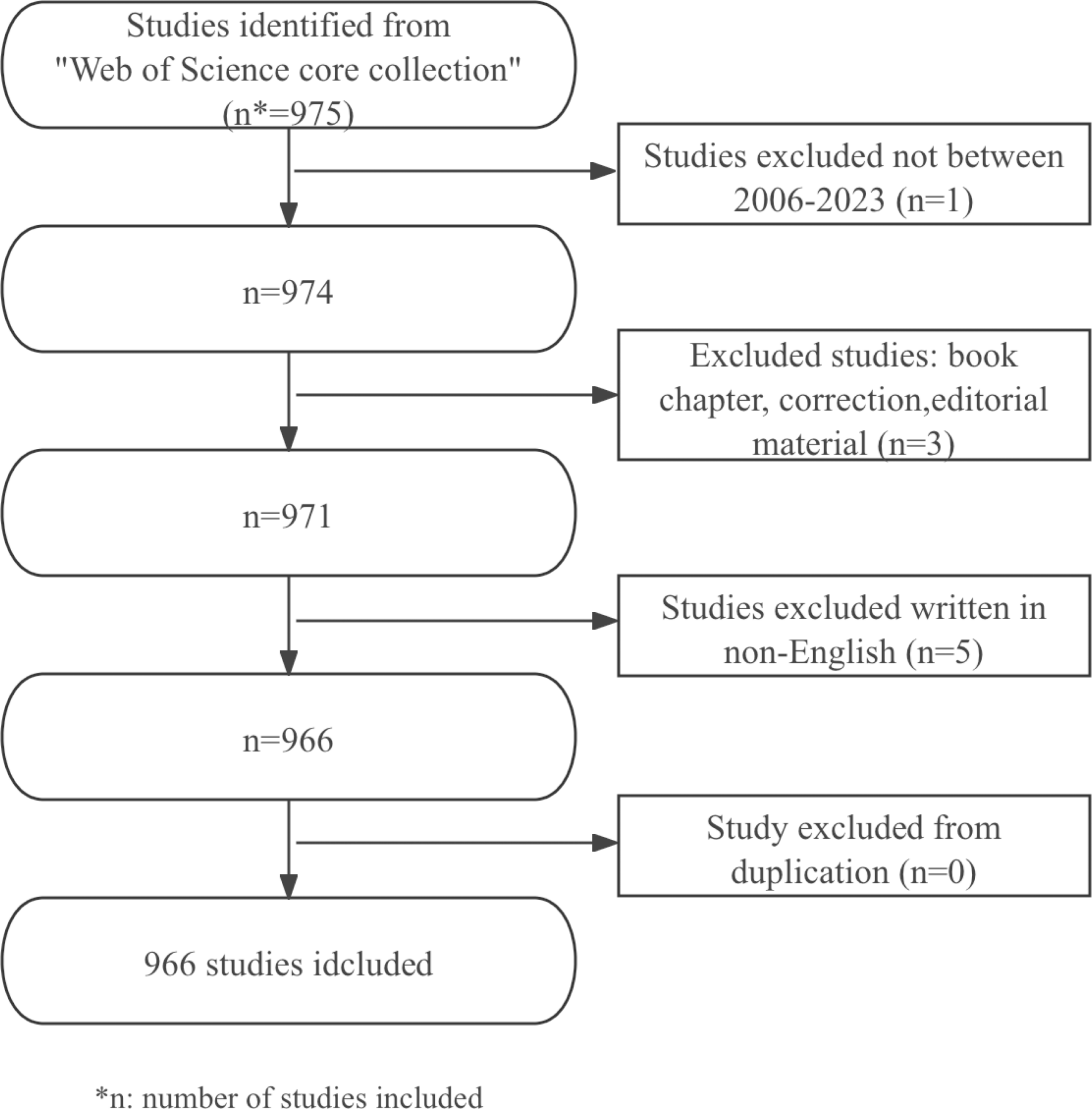
Strategy of data collection and retrieval.

This study included literature on nanotechnology and lung cancer diagnosis published in different English-language academic journals, including articles and review articles. The exclusion criteria were: meeting abstracts, conference presentations, letters, repeated publications, and unrelated articles. Two reviewers independently sifted through the literature and data. After screening, a total of 966 usable articles were obtained. The data collection and retrieval strategy were shown in **Figure 1**.

### 2.3 Software analysis

The analysis and visualization of the annual publication trends and proportions of national papers in this study rely on GraphPad Prism v9.4.0. Additionally, CiteSpace version 6.2.R4(64-bit) (Drexel University, Philadelphia, PA, USA) and VOSviewer version 1.6.20 (Leiden University, Leiden, Netherlands) were utilized for further analysis of the data and for visualizing the scientific knowledge atlas.

VOSviewer, a free JAVA-based software created by Waltman L and van Eck NJ in 2009, can be used to analyze large volumes of literature data and visualize it in map format (18). VOSviewer is a software tool designed for constructing and visualizing bibliometric networks. It has been widely used in the fields of bibliometrics and scientometrics to analyze and visualize relationships among scientific publications, authors, institutions, and keywords. VOSviewer enables users to create visual representations of various types of networks, including citation networks, co-authorship networks, and keyword co-occurrence networks. These visualizations help to illustrate the connections and relationships between different scientific entities. By analyzing bibliometric data, VOSviewer can help identify research trends, emerging fields, and influential publications or authors. The software can identify clusters of related publications, authors, or keywords, providing insights into how particular themes or areas of study are connected within the broader context of scientific research. In this study, we applied VOSviewer 1.6.20 to conduct network diagram and density map of the number of article publishment in journals and the keywords.

CiteSpace, developed based on Java, is a bibliometrics software to visualize research achievements in a particular field through drawing co-citation network maps (19). The software aims to use an experimental framework to study new concepts and evaluate existing technologies, enabling users to gain a better understanding of knowledge domains, research frontiers, trends, and predict future research advancements. CiteSpace allows users to perform comprehensive bibliometric analyses, helping to evaluate citation patterns, authorship trends, and the influence of specific publications within a given field. CiteSpace can create co-citation networks that show how often two or more documents are cited together, as well as co-authorship networks that display collaborations between researchers. This feature aids in understanding collaborative research dynamics. In addition, the tool provides temporal visualizations that highlight trends and changes over time within a specific research area. CiteSpace is equipped to identify emerging research frontiers by analyzing keywords and their occurrences in the literature. In this study, we utilized CiteSpace 6.2.R4(64-bit) to conduct cluster analysis and collaborative network analysis of authors, institutions, journals, literature and countries/regions.

## 3 Results

The results show that from January 1, 2006, to December 14, 2023, a total of 966 articles on the application of nanoparticles in lung cancer were found in the WoSCC database. This includes 711 (73.6%) articles and 255 (26.4%) reviews. The literature covers 73 countries and regions, 1552 institutions, and 5240 authors.

### 3.1 Analysis of annual published papers

**Figure 2:**
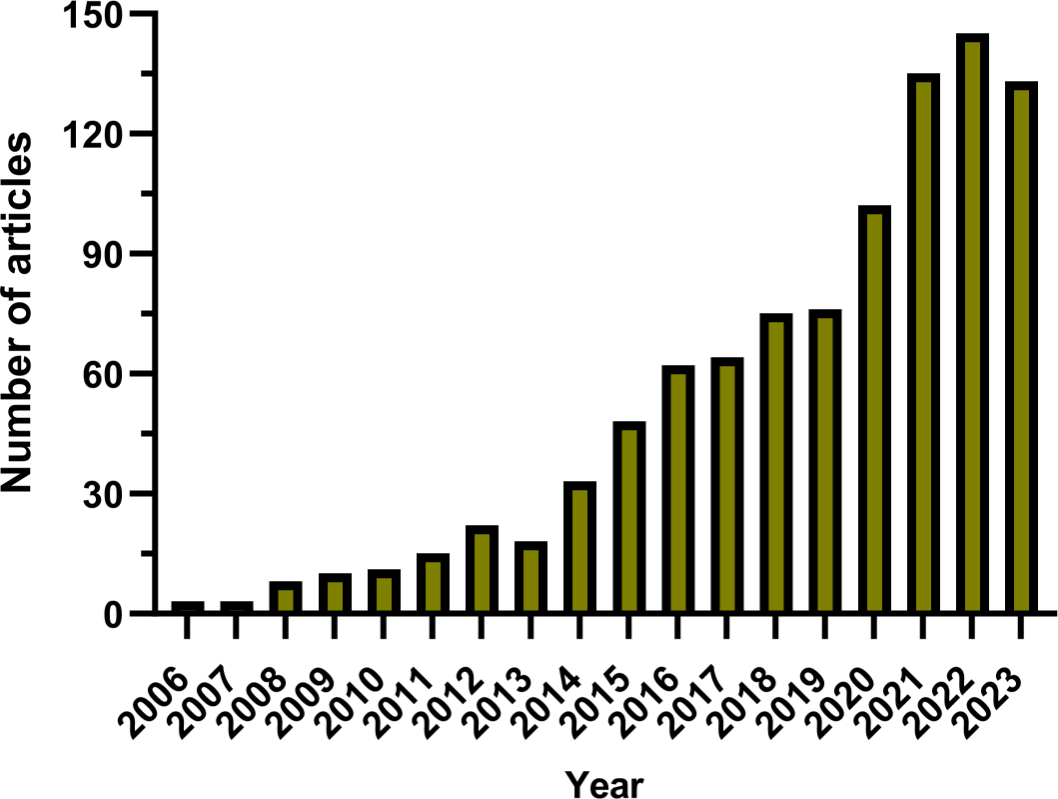
The annual number of global publications.

Since 2006, there has been a gradual increase in the annual publication count within the domain, as illustrated in **Figure 2**. This timeline has been segmented into three distinct phases for analytical clarity. The initial phase, from 2006 to 2013, exhibited a modest growth in publications, with fewer than 30 papers being published annually, reflecting limited engagement from the research community. The subsequent phase, from 2014 to 2019, was characterized by a steady uptick in publication numbers, denoting an escalating interest and visibility of the field within the academic circles. The period following 2020 marked a significant surge in publication volume, culminating in a peak in 2022, which signifies a broad recognition and intensive exploration of the field post-2020. Overall, the utilization of nanoparticles in lung cancer diagnostics is garnering escalating interest and is under constant advancement.

### 3.2 Global publications and cooperation analysis of different countries/regions and institutions

**Figure 3:**
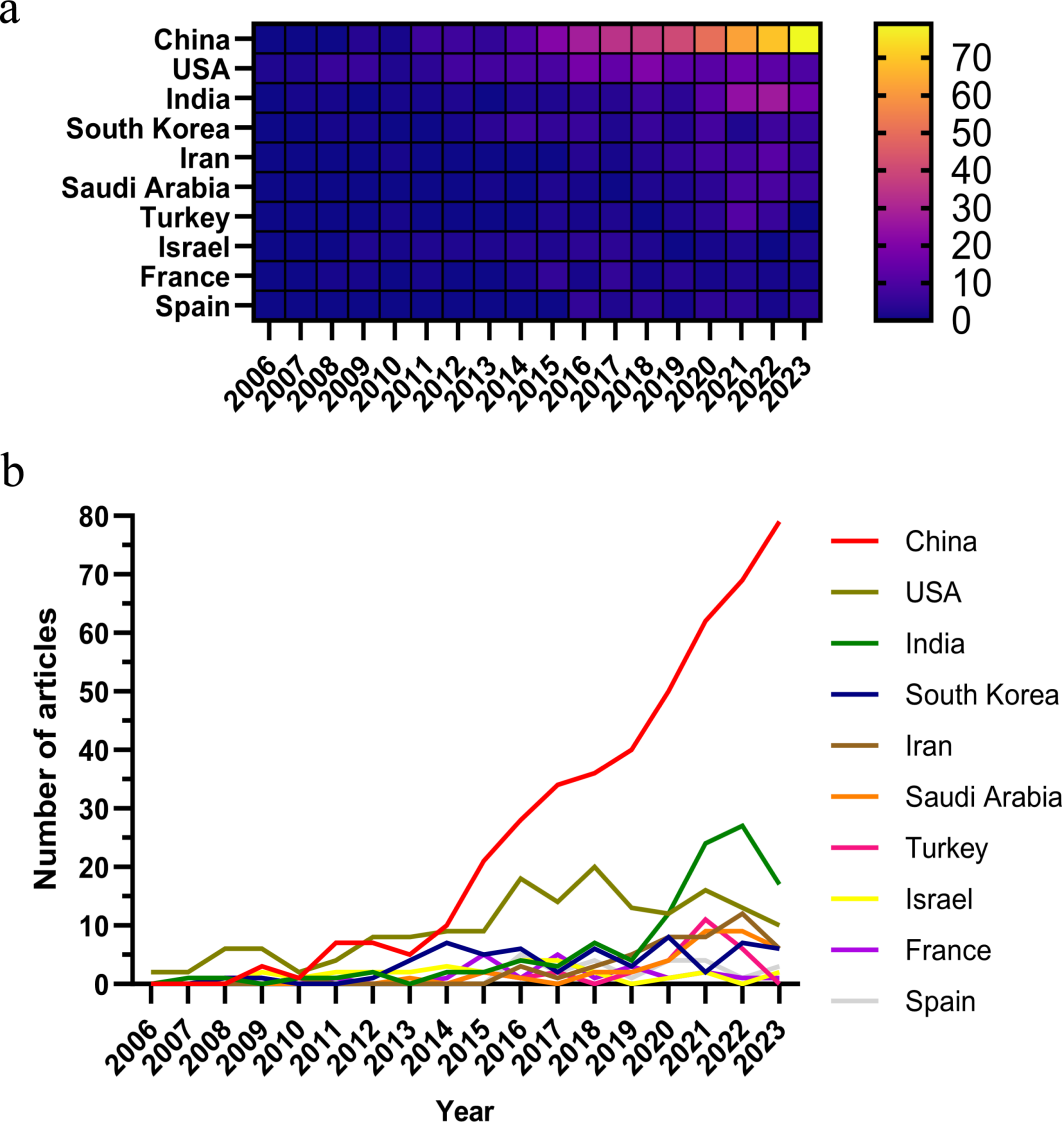
Heat map **(A)** and Line chart **(B)** of national publication volume.

As of the specified data collection deadline, investigations into the application of nanoparticle for lung cancer diagnostics have been conducted in 73 distinct countries and regions. **Table 1** shows the top 10 countries/regions ranked according to their publication volume, and the top five countries in this field are China, the United States, India, South Korea, and Iran. China contributed the most published papers (452, 46.12%), followed by the USA (172, 17.55%), and India (108, 11.02%). The heat map and line chart plotted by the number of publications issued by each country are shown in **Figure 3**. The number of publications of the USA has remained at a dozen, with a peak occurring in 2018 (20), while India’s publications peaked in 2022 (27). In contrast, China’s publications have been surging since 2015, and as of the present, China has already published 79 papers in 2023.

**Table 1:** The top 10 countries/regions by publication volume.

Rank	Country/Region	Article counts	centrality	Percentage (%)	Citation	Citation per publication
1	China	452	0.35	46.12	12827	28.38
2	USA	172	0.23	17.55	12405	72.12
3	India	108	0.19	11.02	2672	24.74
4	South Korea	59	0.03	6.02	2938	49.80
5	Iran	47	0.17	4.80	1215	25.85
6	Saudi Arabia	36	0.13	3.67	617	17.14
7	Israel	29	0.03	2.96	3646	125.72
8	Turkey	29	0.02	2.96	949	32.72
9	Spain	24	0.18	2.45	982	40.92
10	France	24	0.02	2.45	1618	67.42

Among the top 10 countries/regions by publication volume, China and the United States stand out significantly, with their papers receiving 12,827 and 12,405 citations respectively, surpassing the other nations by a wide margin. China leads in both publication volume and the number of citations, yet its citation per publication ratio is 28.38, ranking only seventh. Conversely, the United States, securing the second position in terms of both publication volume and citation count, boasts a citation per publication ratio of 72.12, which is the second-highest, reflecting a superior overall quality of their research outputs.

The institutions ranked in top 10 for publication volume are as shown in **Table 2**. A total of 1552 institutions systematically published articles on the application of nanoparticle in diagnosis of lung cancer. In terms of the leading research institutes, The Chinese Academy of Sciences topped the list with 59 publications and 1759 citations, while Shanghai Jiao Tong University (39 publications and 1287 citations) and Fudan University (18 publications and 688 citations) were the top three institutions in terms of the highest number of publications in China, holding the top four positions. The Technion Israel Institute of Technology in Israel held the third position with 27 publications, however, its notable citation frequency of 3,601 resulted in an impressive average citation of 133.37, positioning it at the forefront in terms of citation impact.

**Table 2:** The top 10 institutions by publication volume.

Rank	Institution	Country	Number of studies	Total citations	Average citation
1	Chinese Academy of Sciences	China	59	1759	29.81
2	Shanghai Jiao Tong University	China	39	1287	33.00
3	Technion Israel Institute of Technology	Israel	27	3601	133.37
4	Fudan University	China	18	688	38.22
5	Indian Institute of Technology System (IIT System)	India	17	590	34.71
6	University of Chinese Academy of Sciences	China	17	871	51.24
7	Nanjing University	China	15	531	35.40
8	Nanjing Medical University	China	15	289	19.27
9	Tongji University	China	14	354	25.29
10	Harvard University	USA	13	638	49.08

**Figure 4:**
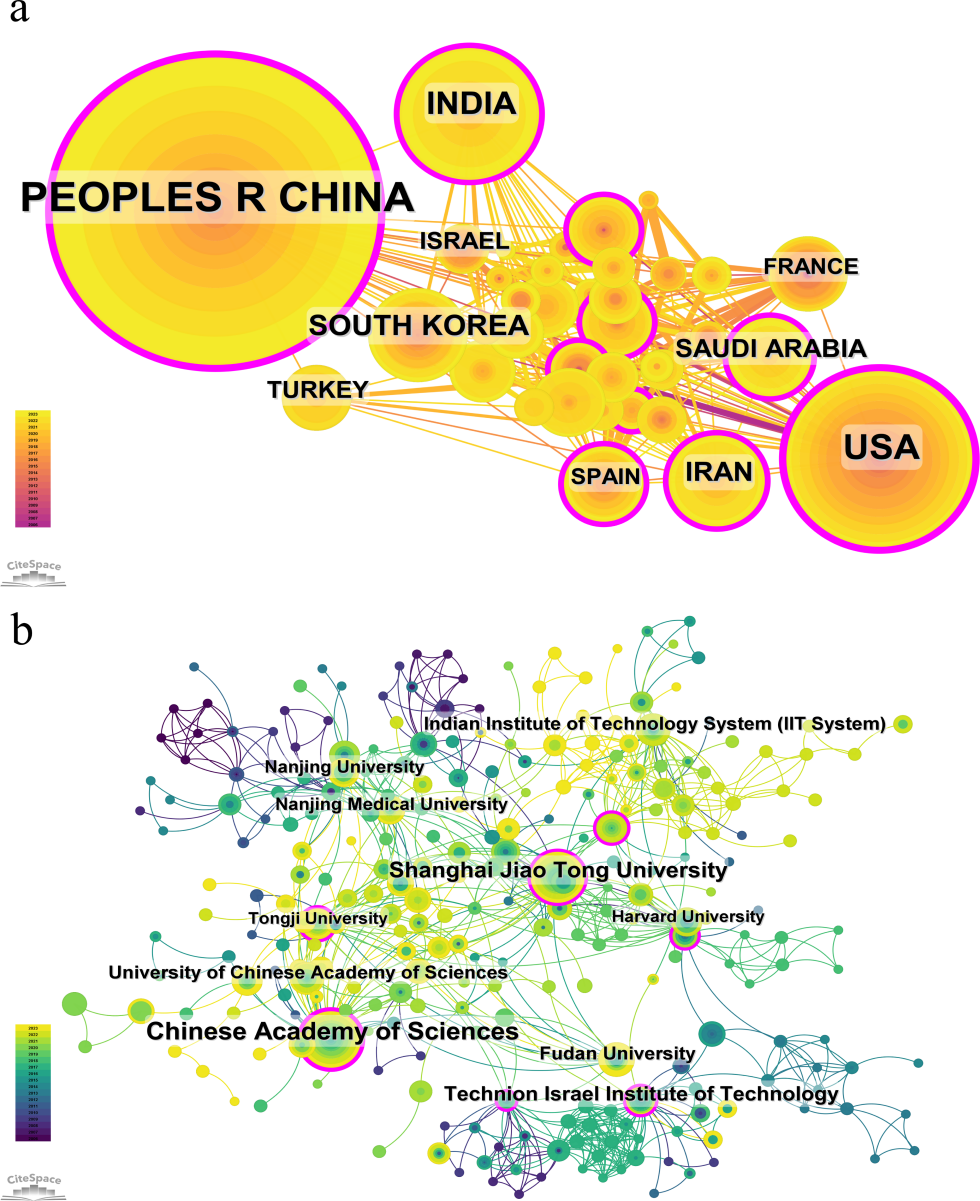
Cooperation analysis of different countries/regions **(A)** and institutions **(B)**.

**Figure 4** presents the cooperation analysis of different countries/regions and institutions. As shown in the picture, China have a large number of publications and high citation frequency, while its centrality index is 0.35, indicating that it is the leading country in this field at present. China cooperates more closely with India, South Korea, Turkey, and Israel, while the United States cooperates more closely with Iran, France, and Spain. This indicates academic cooperation between countries exhibits regional characteristics. Upon further analysis, we discovered that research institutions, regardless of country, have a propensity for collaborating with institutions situated within their respective countries. As a result, we advocate for an intensification of cooperation among institutions at both the domestic and international levels to eradicate the barriers obstructing academic collaboration.

### 3.3 Publications, cooperation and co-citation analysis of journals and authors

**Figure 5:**
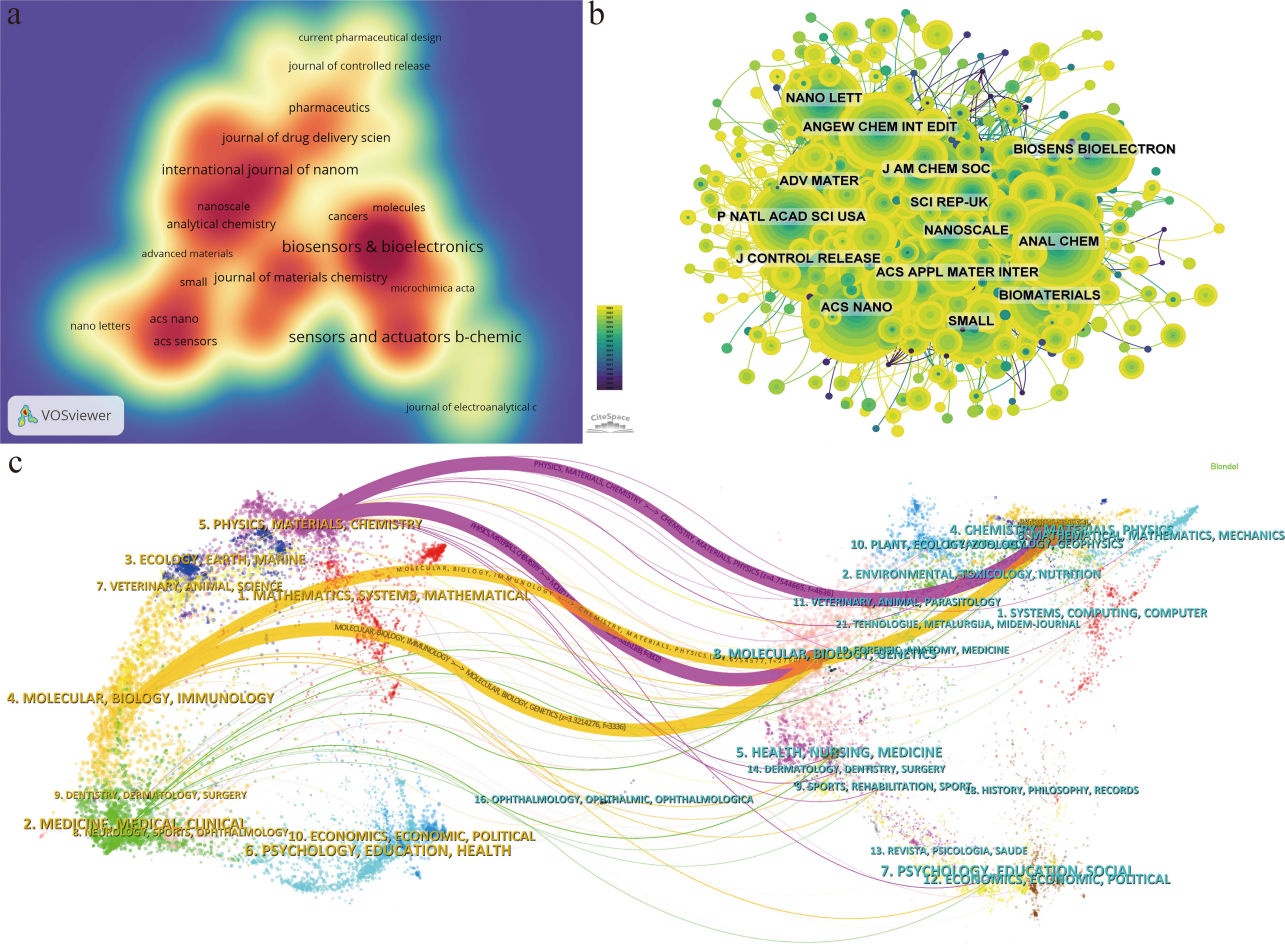
**(A)** Journal density map based on the publications. **(B)** Visualization map of journals co-citation. **(C)** Overlay graph of subject distribution.

**Table 3** presents the top 10 journals by publication volume, as illustrated by the density map in **Figure 5A**. The *Biosensors & Bioelectronics* (34 articles, 3.52%) is the journal with the greatest quantity of publications in this field, followed by *Sensors and Actuators B-Chemical* (31 articles, 3.21%), *ACS Applied Materials & Interfaces* (21 articles, 2.17%), and *International Journal of Nanomedicine* (21 articles, 2.17%). All top 10 publication journals are classified in JCR-Q1, with biosensors & bioelectronics having the highest IF (12.6).

**Table 3:** The top 10 journals by publication volume.

Rank	Journal	Article counts	Percentage (966)	IF	Quartile in category
1	biosensors & bioelectronics	34	3.52	12.6	Q1
2	sensors and actuators b-chemical	31	3.21	8.4	Q1
3	acs applied materials & interfaces	21	2.17	9.5	Q1
4	international journal of nanomedicine	21	2.17	8.0	Q1
5	journal of drug delivery science and technology	16	1.66	5.0	Q1
6	talanta	16	1.66	6.1	Q1
7	journal of materials chemistry b	15	1.55	7.0	Q1
8	acs sensors	14	1.45	8.9	Q1
9	analytica chimica acta	14	1.45	6.2	Q1
10	journal of nanobiotechnology	14	1.45	10.2	Q1

The impact of a journal is determined by the frequency that was cited by others, indicating whether the journal has had a significant impact in this field. The top 10 co-citation journals are exhibited in **Table 4**, and **Figure 5B** shows the visualization map of journals co-citation. There were five journals with more than 350 co-citations, and all of them were in JCR-Q1. *ACS Nano* was the most frequently co-cited journal (488 times), followed by the *ACS Applied Materials & Interfaces* (419 times), the Journal of the American Chemical Society (399 times), the *Biomaterials* (396 times), and the *Analytical Chemistry* (381 times), while the *Biomaterials* in the 5th place had a greater centrality and the *Advanced Materials* in the 9th place possessed a higher IF (29.4). It demonstrated that Biomaterials played an important role in the development of this subject area.

**Table 4:** The top 10 journals by co-citation.

Rank	Cited Journal	Co-Citation	IF (2022)	Quartile in category
1	ACS NANO	488	17.1	Q1
2	ACS APPL MATER INTER	419	9.5	Q1
3	J AM CHEM SOC	399	15.0	Q1
4	BIOMATERIALS	396	14.0	Q1
5	ANAL CHEM	381	7.4	Q1
6	NANOSCALE	349	6.7	Q1
7	P NATL ACAD SCI USA	348	11.1	Q1
8	SMALL	338	13.3	Q1
9	ADV MATER	333	29.4	Q1
10	SCI REP-UK	330	4.6	Q2

The distribution of topics in academic publications is displayed through dual overlap map (**Figure 5C**). The colored lines represent the connections between citations, with citing domains on the left and cited domains on the right. Based on the displayed results, we identified 4 main colored citation paths, namely that research publications in the fields of physics/materials/chemistry being cited mainly by research publications in the fields of molecular/biology/genetics and chemistry/materials/physics. Research publications in the field of molecular/biology/immunology is mainly cited by research publications in the fields of molecular/biology/genetics and chemistry/materials/physics. It can be seen that the application of nanoparticle in lung cancer diagnosis involves several main realms such as materials, chemistry, biology, molecular, and immunology.

**Figure 6:**
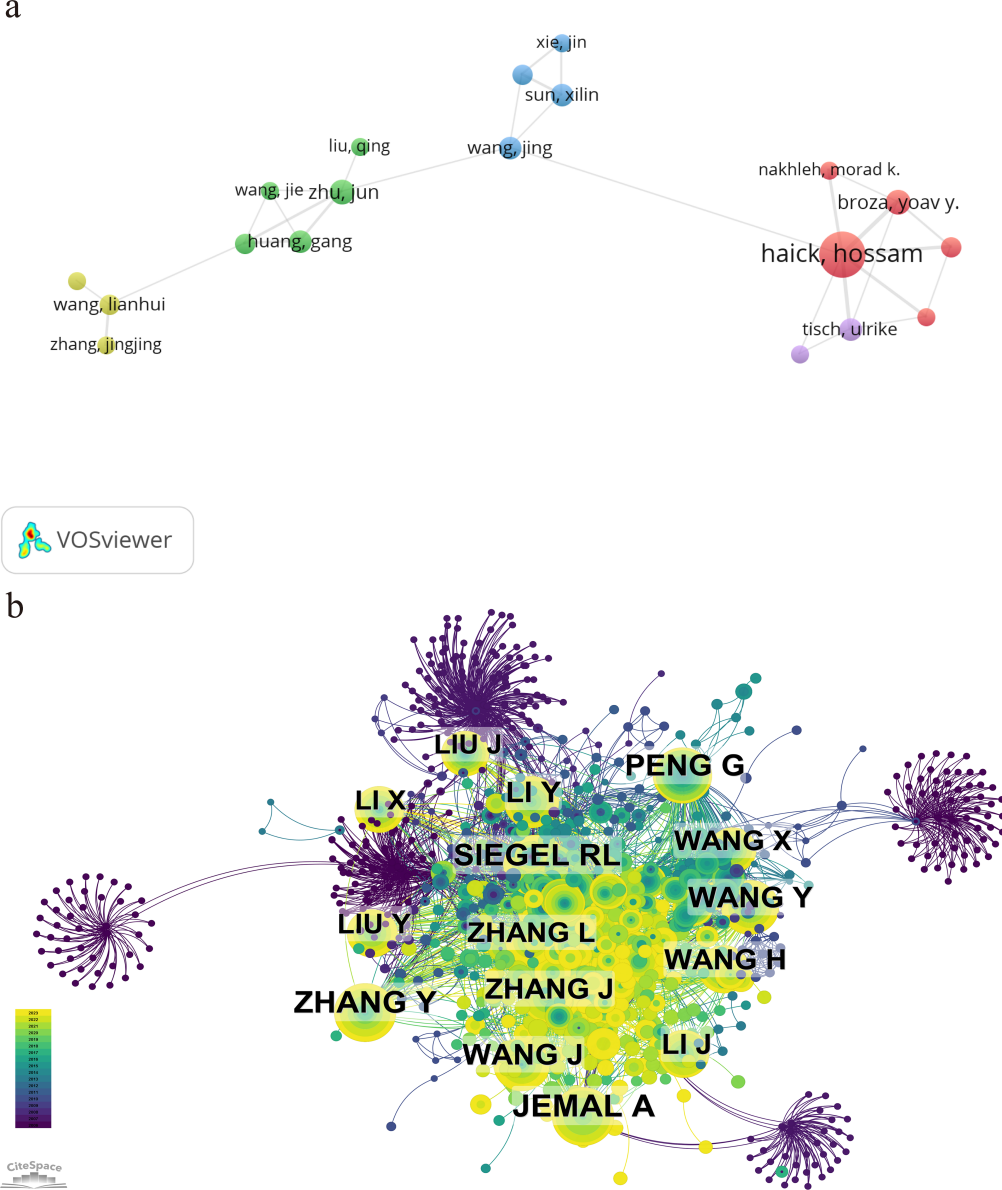
Network map based on cooperation between authors **(A)** and authors co-citation **(B)**.

**Table 5** represents the top 10 authors with the greatest number of publications and co-citations, respectively. The top 10 authors published a total of 98 papers, accounting for 10.14% of all articles in the field. Haick, Hossam has the largest quantity of publications (24 articles), followed by Cui, Daxiang (11 articles) and Yang, Huaixia (10 articles). Among the top 10 ranked authors, six are from China, two from Israel, and two from South Korea. The network map based on cooperation between authors is shown in **Figure 6A**. A knowledge map of author co-citation analysis was displayed in **Figure 6B**. There are a total of 16 authors who have been cited over 50 times, indicating that the results published by these researchers had had a strong impact on the realm that the application of nanoparticle in lung cancer diagnosis. Among them, Jemal, A, with the biggest node, was the most cocited author (111 times), followed by Zhang, Y (98 times), Siegel, RL (95 times), Peng, G (90 times), and Wang, J (79 times). The above data revealed that Jemal, A held a prominent position in the realm of application of nanoparticle in lung cancer diagnosis.

**Table 5:** The top 10 authors by publications and co-citation.

Rank	Author	Count	Location	Rank	Co-cited author	Citation
1	Haick, Hossam	24	Israel	1	Jemal, A	111
2	Cui, DaXiang	11	China	2	Zhang, Y	98
3	Yang, HuaiXia	10	China	3	Siegel, RL	95
4	Kim, IL-Doo	9	Korea	4	Peng, G	90
5	Cao, XiaoWei	8	China	5	Wang, J	79
6	Choi, Seon-Jin	8	Korea	6	Wang, Y	79
7	Kong, JinMing	8	China	7	Li, Y	74
8	Broza, Yoav y.	7	Israel	8	Wang, H	71
9	Zhu, Jun	7	China	9	Li, J	67
10	Dong, Jian	6	China	10	Zhang, J	67

### 3.4 Visualization and cluster analysis of co-cited references

**Figure 7:**
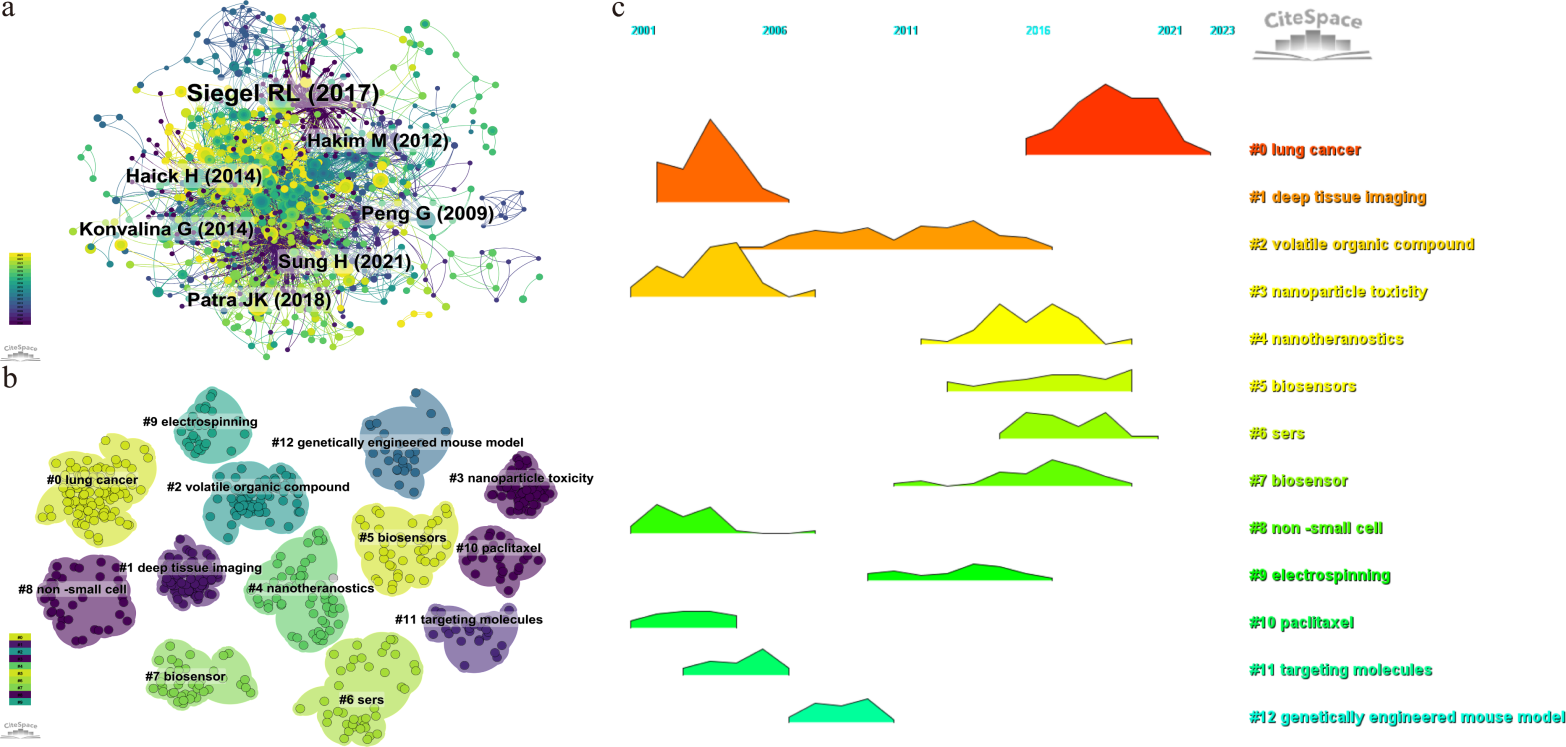
Visualization map of co-cited references. **(A)** the co-occurrence visualization network of co-cited references. **(B)** cluster analysis of co-cited references. **(C)** volcano map of cluster analysis.

By setting the selection criteria as g-index(k=25), LRF=3.0, L/N=10, LBY=5, and e=1.0, the co-cited reference co-occurrence visualization network consisting of 995 nodes and 3346 connections in **Figure 7A** was obtained, in which the size of the nodes represented the frequency, and the thickness of the lines represented the closeness of the connection. In this figure, the color of the nodes represents the year of publication, with larger nodes indicating that the article has been cited more frequently. The greater the number of connecting lines, the higher the recognition of the article within the field. The article “Cancer Statistics, 2021” in the *CA: A Cancer Journal for Clinicians* (IF=254.7) is the reference with the highest number of co-citations, with Siegel, Rebecca L. as the first author. The American Cancer Society annually estimates the number of new cancer cases and deaths in the United States and compiles the latest data on population-based cancer occurrence. This article expresses that the mortality rate of cancer, especially lung cancer, is decreasing year by year, with the annual decline rate of lung cancer mortality in male having increased from 3.1% to 5.5%, while that in female having increased from 1.8% to 4.4%, and overall mortality rate from 2.4% to 5%. The 2-year relative survival rate of NSCLC has increased from 34% to 42%, while the survival rate of small cell lung cancer remains 14% - 15%. The innovation of diagnostic and treatment methods has accelerated the development of lung cancer treatment, especially the application of nanoparticles, which has opened up a new avenue for the diagnosis and treatment of NSCLC, and making a series of progress, promoting the reduction of the overall mortality rate of cancer.The second article is “Assessment, origin, and implementation of breath volatile cancer markers”, published by Haick,Hossam, which proposes that cancer can be diagnosed by detecting volatile organic compounds (VOCs) in exhaled air samples, introducing a new method that is non-invasive and potentially cost-effective. Breath analysis is a very young field of research and faces challenges. Nanoparticles can be applied to perform targeted analysis of VOCs related to cancer. The visual map of co-cited references cluster analysis presented in **Figure 7B** is obtained by log-likelihood ratio (LLR) algorithm, and the details were listed in **Table 6**. The top 12 clusters included #0 lung cancer, #1 deep tissue imaging, #2 volatile organic compounds, #3 nanoparticle toxicity, #4 nanotheranostics, #5 biosensors, #6 SERS(Surface Enhanced Raman Scattering), #8 NSCLC(Non-small Cell Lung Cancer), #9 electrospinning, #10 paclitaxel, #11 targeting molecules, and #12 genetically engineered mouse model. **Figure 7C** shows that deep tissue imaging(cluster 1), nanoparticle toxicity(cluster 3), NSCLC(cluster 8), paclitaxel(cluster 10) are early research hotspots, and volatile organic compound(cluster 2), electrospinning(cluster 9), targeting molecules(cluster 11), genetically engineered mouse model(cluster 12) are mid-term hotspots, and nanotheranostics(cluster 4), biosensors(cluster 5), SERS(cluster 6) represent the hot topics and trends in the field. The horizontal axis represents the year, and a higher shape indicates a greater level of prominence for the topic.

**Table 6:** The top 10 references with most co-citations.

Rank	Title	Journal & IF (2023)	author (s)	Total citations
1	Cancer Statistics, 2021	*CA-A CANCER JOURNAL FOR CLINICIANS* (IF=254.7)	Siegel RL	40
2	Assessment, origin, and implementation of breath volatile cancer markers	*CHEMICAL SOCIETY REVIEWS* (IF=46.2)	Haick H	18
3	Nano based drug delivery systems: recent developments and future prospects	*JOURNAL OF NANOBIOTECHNOLOGY* (IF=10.2)	Patra JK	18
4	Diagnosing lung cancer in exhaled breath using gold nanoparticles	*NATURE NANOTECHNOLOGY* (IF=38.3)	Peng G	17
5	Global cancer statistics 2020: GLOBOCAN estimates of incidence and mortality worldwide for 36 cancers in 185 countries	*CA-A CANCER JOURNAL FOR CLINICIANS* (IF=254.7)	Sung H	17
6	Sensors for Breath Testing: From Nanomaterials to Comprehensive Disease Detection	*ACCOUNTS OF CHEMICAL RESEARCH* (IF=18.3)	Konvalina G	16
7	Volatile Organic Compounds of Lung Cancer and Possible Biochemical Pathways	*CHEMICAL REVIEWS* (IF=62.1)	Hakim M	16
8	Detection of lung, breast, colorectal, and prostate cancers from exhaled breath using a single array of nanosensors	*BRITISH JOURNAL OF CANCER* (IF=8.8)	Peng G	15
9	Nanomaterial-based sensors for detection of disease by volatile organic compounds	*NANOMEDICINE* (IF=5.5)	Broza YY	15
10	Classification of lung cancer histology by gold nanoparticle sensors	*NANOMEDICINE-NANOTECHNOLOGY BIOLOGY AND MEDICINE* (IF=5.4)	Barash O	14

### 3.5 Visualization and cluster analysis of key words

**Figure 8:**
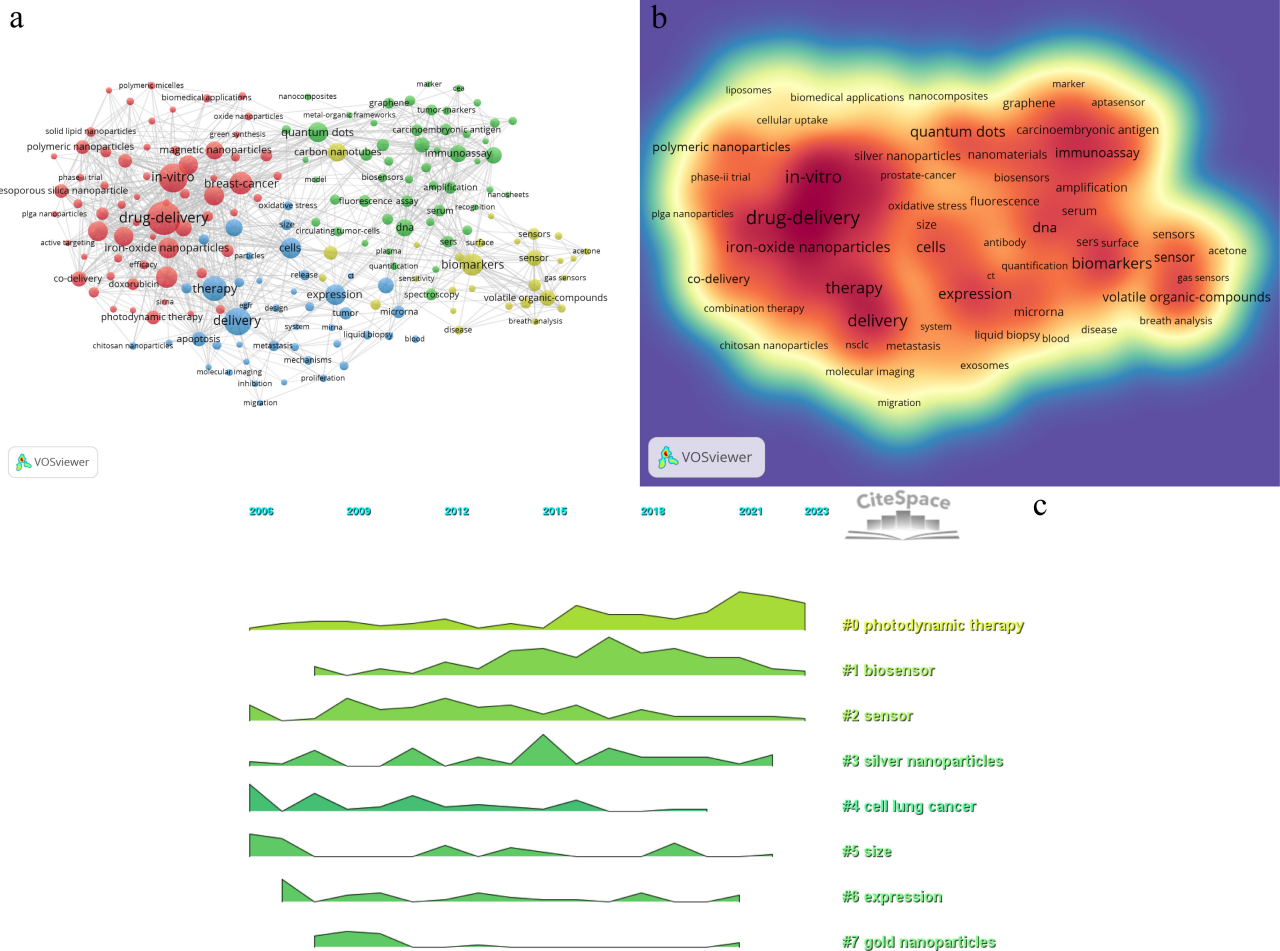
Visualization map of key words. **(A)** the co-occurrence visualization network of key words. **(B)** heat map of key words. **(C)** volcano map of cluster analysis.

**Table 7** listed the top 10 keywords by frequency and link strength, and their visual maps are respectively shown in **Figures 8A** and **8B**. According to the co-occurrence of keywords in VOSviewer, and as the frequency of co-occurrence is indicative of its popularity, “drug-delivery”, “*in-vitro*”, “delivery”, “therapy”, “breast-cancer”, “biomarkers”, “chemotherapy”, “cells”, “expression”, and “iron-oxide nanoparticles” had been identified as top 10 hot keywords. This suggests that current research on nanoparticles in lung cancer predominantly concentrate on their application in drug delivery, with the objective of utilizing this approach for therapeutic interventions. We removed irrelevant keywords and constructed a network of 182 keywords which appeared at least 28 times, resulting in four different clusters. The first cluster is red, with 63 keywords, including *in-vitro*、drug-delivery、chemotherapy、nanomedicine、tumor microenvironment、magnetic nanoparticles, targeted delivery, co-delivery, photodynamic therapy, imaging; the second cluster is green, with 52 keywords, including DNA, SERS, biosensor, immunoassay, quantum dots, sensitive detection, spectroscopy, aptamer, fluorescence, amplification, assay, platform, graphene; the third cluster is blue, with 43 keywords, including delivery, therapy, cells, expression, biomarker, cytotoxicity, apoptosis, resistance, metastasis, exosm, activation, migration, oxidative stress; and the fourth cluster is yellow, with 28 keywords, including biomarkers, carbon nanotubes, gas sensors, breath, array, surface, disease, acetone. We utilized CiteSpace to create a volcano plot, thereby visually illustrating the temporal evolution of research hotspots (**Figure 8C**). The figure reveals that “photodynamic therapy”, “biosensors”, and “silver nanoparticles” have remained consistently prominent research themes in this domain.

**Table 7:** The top 10 keywords by frequency and link strength.

Rank	Keyword	Counts	Keyword	Link strength
1	drug-delivery	110	drug-delivery	539
2	*in-vitro*	90	*in-vitro*	459
3	delivery	83	delivery	332
4	therapy	72	nanomedicine	296
5	breast-cancer	64	nanotechnology	280
6	biomarkers	61	quantum dots	279
7	chemotherapy	59	breast-cancer	278
8	cells	55	biomarkers	271
9	expression	55	therapy	269
10	iron-oxide nanoparticles	55	iron-oxide nanoparticles	267

### 3.6 Strongest citation burst of references and key words

**Figure 9:**
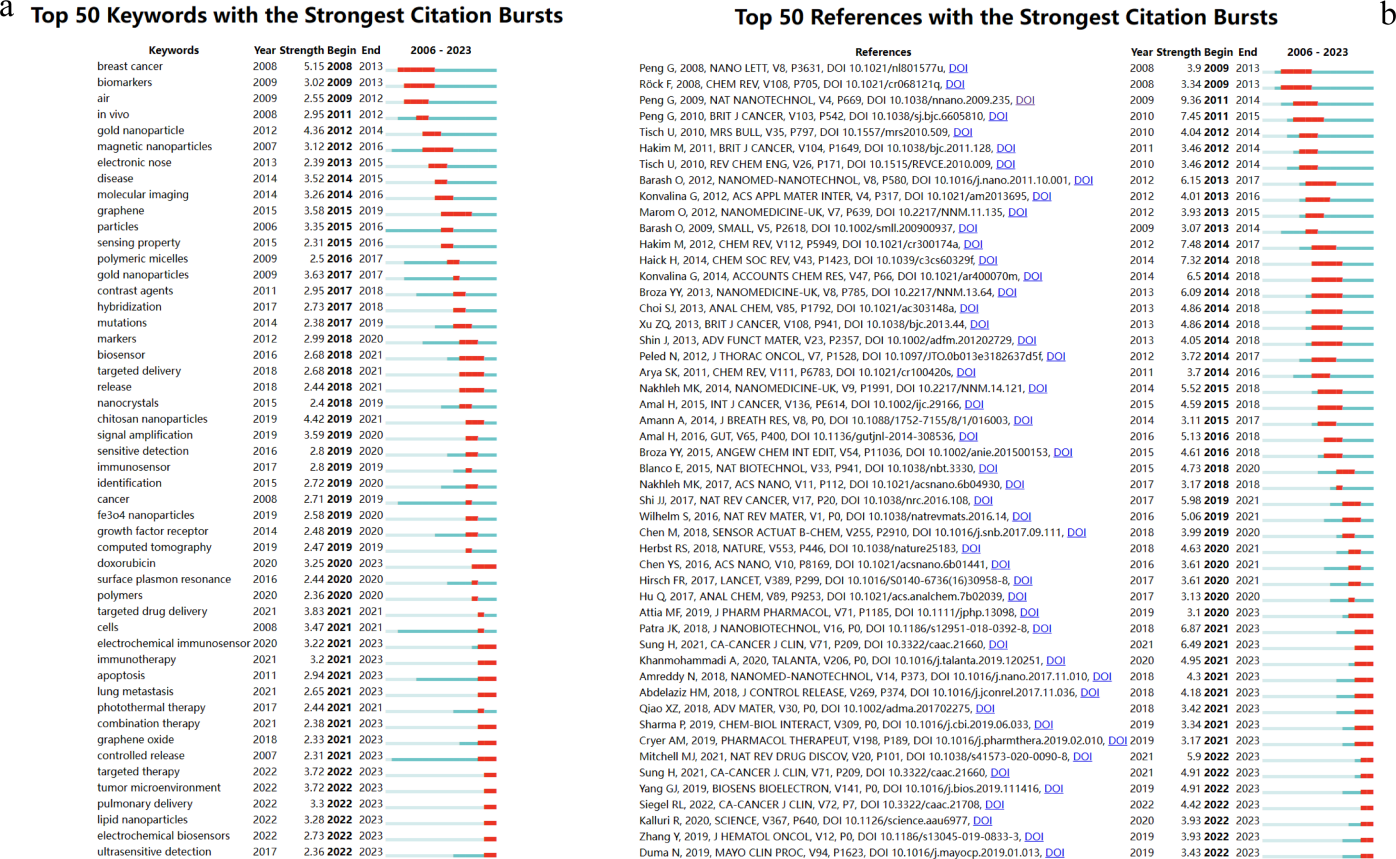
Top 50 **(A)** key words and **(B)** references with the strongest citation bursts.

Burst analysis is a method that identifies research hotspots and emerging frontiers within a specific period. **Figures 9** illustrate the intensity of these bursts, along with their respective start and end times. These visualization diagrams are generated by CiteSpace.

**Figure 9A** enumerates the top 50 keywords exhibiting the most pronounced citation bursts. The initial phase of research predominantly centered on “biomarkers” (2009-2013), “gold nanoparticles” (2012-2014), and “magnetic nanoparticles” (2012-2016). This trend underscores an early emphasis on the application of nanomaterials and biomarker detection. In subsequent years, the emergence of terms such as “chitosan nanoparticles,” “targeted drug delivery,” “targeted therapy,” and “tumor microenvironment” illustrates a shift towards a more nuanced focus on the tumor microenvironment and targeted therapeutic delivery. This evolution highlights the burgeoning synergy between nanomaterials and biotechnology as a pivotal area of research. Furthermore, the dynamic progression of research themes over time attests to the ongoing advancements in nanomaterial technologies and innovative strategies for lung cancer diagnosis, indicating a positive developmental trajectory in this domain.

In terms of the top 50 references exhibiting the most significant citation bursts, Peng G’s 2009 publication in *Nature Nanotechnology* and a 2010 paper in the *British Journal of Cancer*, along with Blanco E’s 2015 publication in *Nature Biotechnology*, and Amreddy N’s 2018 paper in *Nanomedicine: Nanotechnology, Biology, and Medicine*, have covered a period of time until 2023. The research content in these articles is consistent with the key concerns reflected in the current burst keywords(**Figure 9B**).

## 4 Discussion

Over the past nearly two decades, the number of studies on nanoparticles in the field of lung cancer diagnosis has continuously increased, exhibiting an overall upward trend. From 2006 to 2013, research on nanoparticles in lung cancer diagnosis was in its nascent stages, with an average annual publication fewer than 30 articles. Subsequently, from 2014 to 2023, research in this field has been significantly strengthened, with an average annual publication 89.1 articles, peaking in 2022 with 145 articles. This trend indicates a growing involvement of researchers in the study of nanoparticles for lung cancer diagnosis. Notably, China leads in publication number, contributing 452 articles, accounting for 46.79%.

The number of research papers in this field is concentrated in economically strong countries. The h-index and the number of citations are frequently utilized to evaluate the academic standing of a country or region (20–22). The United States pioneered the application of nanomaterials in lung cancer diagnosis and has yielded prolific research outcomes. Between 2006 and 2010, more than half (52.9%) of the publications were published by them. Over the past decade, starting from 2014, China has experienced a significant surge in publication number, reflecting increased research investment in the application of nanomaterials in lung cancer diagnosis and expanding collaboration with various research institutions both domestically and internationally, thereby propelling the global advancement of this field. Extensive international scientific collaboration contributes to the maturation of the research field (23). However, the citation-to-publication ratio of Chinese papers ranks only seventh among all countries, suggesting that the quality of research still requires enhancement, indicating a discrepancy between quantity and quality.

Regarding institutional contributions, the Chinese Academy of Sciences and Shanghai Jiao Tong University rank first and second in publication number, respectively. However, the Technion Israel Institute of Technology, which ranks third, boasts the highest total citation volume and average citation volume, signifying that its research in this field is widely acknowledged and esteemed worldwide.

After excluding irrelevant keywords, we constructed a network comprising 182 keywords, each appearing at least 28 times, thereby delineating four distinct clusters. Notably, “biomarkers” emerged as the sixth most frequent keyword and constitutes a pivotal element of the yellow cluster, intricately associated with other terms, thereby underscoring its indispensable role in early cancer diagnosis. Biomarkers have persistently been a focal point in lung cancer diagnostics. The oncogenic process is frequently accompanied by mutations in DNA and RNA, aberrant protein expression, and methylation or point mutations of organic compounds such as cytokines. Many of these alterations can be detected months or even years before clinical diagnosis, and are thus identified as cancer biomarkers (24–26). Consequently, utilizing cancer biomarkers for preventive lung cancer diagnosis in high-risk cohorts can preclude the physical detriments associated with radiological examinations and pathological biopsies. Moreover, this approach mitigates the secondary trauma inflicted by invasive tests.

Comprehensive research reveals that biomarkers are predominantly classified into two categories: protein and genetic biomarkers (27). Various cancer biomarkers have been identified for lung cancer detection, including carcinoembryonic antigen (CEA) (28), cytokeratin fragment 21-1 (CYFRA21-1) (29), carbohydrate antigen 125 (CA125) (30), transthyretin (TTR) (31), haptoglobin (32), neuron-specific enolase (NSE) (33), GM2 activator protein (GM2AP) (34), carbohydrate antigen 19-9 (CA19-9) (35), p16 (36), and the KRAS (37).

Among these, CYFRA21-1 and NSE are extensively utilized for lung cancer diagnosis. Moreover, these biomarkers serve as differentiators between non-small cell lung cancer (NSCLC) and small cell lung cancer (SCLC), significantly aiding in the precise diagnosis and classification of lung cancer subtypes (38, 39). Previous studies have corroborated the high specificity and sensitivity of CYFRA21-1 as a biomarker in diagnosing NSCLC, with its efficacy in detecting squamous cell carcinoma being particularly notable (40). Additionally, CYFRA21-1 has demonstrated substantial utility in the therapeutic management of lung cancer patients. Research consistently indicates that CYFRA21-1 levels can predict chemotherapy efficacy in patients with advanced NSCLC. Recent investigations have further substantiated this correlation, revealing that the rate of change in CYFRA21-1 levels before and after the initial chemotherapy cycle inversely correlates with chemotherapy efficacy (41, 42).

Sometimes, a single cancer biomarker may not suffice to achieve a 100% confirmation diagnosis rate for early-stage lung cancer patients; combining multiple lung cancer biomarkers can significantly enhance the diagnostic rate for these patients (43). There are researches have shown that the sensitivity of using a combination of CEA, CYFRA21-1, and NSE as biomarkers is higher than that of using only two or one biomarker. Moreover, the sensitivity of integrating tumor biomarkers with imaging studies for early lung cancer diagnosis reaches up to 90% in clinical case analyses (44). However, the statistical analysis only covered 180 patients, and its reliability needs further validation.

In the keyword clustering volcano plot(**Figure 8C**), “biosensors” emerge as critically important. Researchers and clinicians frequently employ immunological techniques for biomarker analysis, such as the conventional enzyme-linked immunosorbent assay (ELISA), to identify substances like antigens and antibodies. This method is renowned for its exceptional sensitivity and specificity, rendering it an excellent detection tool; however, its complexity and cost restrict its broad applicability (45, 46). Consequently, the development of a portable and rapid biosensor for detecting lung cancer biomarkers could significantly improve early lung cancer diagnosis rates.

Biosensors consist of two primary components: sensors and biometric elements, which capture and react with a series of biomarkers, subsequently converting biochemical reactions into measurable signals to provide clinical personnel with patient testing information expeditiously (38, 47). The performance of biosensors is typically assessed using two metrics: the detection limit and sensitivity. A lower detection limit and higher sensitivity correlate with superior biosensor performance (48). For instance, a recent study reports that immunosensing of NSE, a standard lung cancer biomarker, employs a nanocomposite of mesoporous silica encapsulated with CuO2 nanoparticles to develop an innovative electrochemiluminescence sensing platform. This method shows promise as a cost-effective approach to detecting neuron-specific enolase antigen in serum (49). Additionally, considering the specific site of cancer in the lung, detecting volatile compounds in exhaled breath presents a viable approach, and the development of nano-biosensors could also be targeted towards this research area (50).

Although theoretical research on biosensors is abundant, several challenges persist in their practical design and clinical application. For example, the receptor on the recognition element may rapidly deteriorate, reducing recognition sensitivity. Consequently, the biomarker may not be easily recognized due to alterations when removed from its native environment (51). Therefore, further efforts are necessary for researchers to develop biosensors that can meet the demands of large-scale clinical lung cancer detection.

Nanoparticles, as a distinctive application of nanomaterials in emerging nanotechnology, have become significant research subjects, particularly those nanoparticles utilizing precious metals as the material source. These materials exhibit unique physicochemical properties and possess a high surface area-to-volume ratio, which enhances the detection performance of conventional biosensors (52, 53).

Gold nanoparticles and silver nanoparticles both emerge in the keyword clustering analysis, highlighting their significant roles in lung cancer diagnostics, with “gold nanoparticles” appearing earlier. Many researchers have found that gold nanoparticles are biocompatible due to their chemical inertness. Their high electrical conductivity, density, and surface area-to-volume ratio distinguish them from other materials. Furthermore, the organic combination of gold nanoparticles and biosensors can effectively improve the detection limit and sensitivity of the sensors (54, 55).

Pirzada et al. (56) developed an ultrasensitive electrochemical sensor using AuNPs-modified epitope-mediated hybrid molecularly imprinted polymers (MIP). Experiments showed that AuNPs hybridized MIP improved the sensor’s sensitivity and expanded the detection range to identify NSE in human serum in the concentration range of 25–4000 pg/mL, effectively enhancing the early detection rate of SCLC. Furthermore, Zeng et al. (57) developed an ultrasensitive electrochemical immunosensor to detect CYFRA21-1 in human serum and enhance the screening of NSCLC. The team used AuNPs/Thi/MWCNT-NH2 nanocomposites to immobilize horseradish peroxidase-labeled anti-CYFRA21-1. The high anti-CYFRA 21-1 loading capacity, complemented by the good biocompatibility and conductivity of the nanomaterials, allowed the biosensor to have a high linear range of 0.1–150 ng·mL^-1 and a low detection limit of 43 pg·mL^-1.

In addition to gold nanoparticles, sliver nanoparticles have attracted researchers’ interest for their unique antibacterial properties, thermal stability, electrical conductivity, and catalytic activity. They have applied AgNPs in biosensors’ fabrication to enhance lung cancer biomarkers’ detection limits (58, 59). Lee et al (60). used a mixture of silver nanoparticles and reduced graphene oxide-modified screen-printed electrodes to prepare a sensing matrix. They also used horseradish peroxidase-labeled antibodies as recognition molecules for CEA, resulting in a sandwich-type electrochemical immunosensor that performs better in detecting CEA in a simple, rapid, and low-cost manner. Magnetic nanoparticles can also be used in lung cancer diagnostics (61). However, the current research depth and refinement are insufficient to obtain nanomaterial-derived biosensors with excellent performance.

However, nanoparticles may have potential toxic effects on the human body. Studies have reported that Ag nanoparticles can induce intracellular DNA damage through the GADD45a gene (62). Besides, there is a study shows that the induced DNA damage and activated caspase-3, p53, p38 and ERK expression by Au/Ag NPs offered leads to their higher cytotoxicity and redox modulations (within mitochondrial membranes) (63). The toxicity of Au is often greater than that of Ag. For example, one particular investigation examined the effects of both Au and Ag NPs on A549 cell line at 24-hour interval through which the concerned scientists noted that 29.4 μg/mL as IC50, inhibitory concentration for Ag NPs whereas for Au NPs, this value was 49.8 μg/mL (64). The particle size is one of the factors responsible for showing toxicity of Au NPs, specifically the smaller ones. This is due to their ability to cross the cell membrane and reach the nucleus more rapidly (65). Not only the size, but also the quantity of nanoparticles and whether they are loaded with other biological materials can influence their toxicity (66). Albumin-modified gold nanoparticles and chitosan functionalized silver nanoparticles have been demonstrated to possess low *in vivo* toxicity (67, 68). Furthermore, how to synthesize these nanomaterials and how to remove organic solvents, reagents, or toxic chemicals from the reaction mixture are significant challenges we face (69).

Gold (Au) and silver (Ag) are categorized as inorganic materials, akin to other inorganic nanomaterials such as silica, hydroxyapatite, and similar calcium-based substances. Conversely, organic materials encompass liposomes, polymers, and carbon-containing compounds. These materials are employed for drug loading and improve therapeutic and diagnostic efficacy via targeted delivery mechanisms. Currently, nanoparticle formulations currently approved by the U.S. FDA for clinical trial include (The registration ID in the U.S. clinical trial database):

1. Inorganic substances:

- based on Hafnium oxide (NCT04505267)
- based on gold nanoparticles (NCT01679470)

2. Polymeric micelles nanoparticles:

- based on paclitaxel (NCT01770795, NCT01023347, NCT01792479, NCT02283320)
- based on camptothecin (NCT01380769, NCT01803269)
- based on siRNA (NCT00689065)

Scientists have extensively investigated targeting and permeation, including magnetic nanoparticles (70), thermosensitive nanoparticles (71, 72), and pH-sensitive nanoparticles (73). Simultaneously, scientists have employed tumor cell membranes to encapsulate nanomaterials, enhancing drug delivery efficiency. The aforementioned studies predominantly focus on intravenous administration. Additionally, inhalation therapy emerges as a promising approach for the diagnosis and treatment of lung cancer. Compared to conventional intravenous administration, inhalation therapy provides several advantages, including enhanced pulmonary targeting and reduced systemic drug concentrations, thereby minimizing biological toxicity (74).

The precision targeted therapy for lung cancer based on nanotechnology aims to minimize the toxic effects of drugs, enhance the efficacy of anticancer chemotherapy agents, and improve tumor imaging. In recent years, this research field has significantly expanded, contributing to the improvement of patients’ quality of life and overall survival rates. Nanoparticles, as excellent biomaterials, exhibit diverse properties that make them suitable for drug delivery applications. They provide sufficient space and protection for drug molecules, preserving their integrity during systemic circulation and preventing exposure to non-target tissues. Furthermore, their surfaces can be functionalized with various targeting moieties to selectively target cancer cells and tumors. Inhaled nanoparticle therapy represents a promising treatment strategy. However, inherent cytotoxicity of anticancer drugs raises concerns regarding pulmonary tolerance, as well as the potential risks of local pulmonary toxicity and adverse reactions. In summary, whether through intravenous administration or inhalation, nanoparticles hold great promise in the treatment of lung cancer.

This study is the first to analyze the application of nanoparticles in lung cancer diagnosis using the bibliometrics method, which has certain guidance and pioneering. However, this study has some limitations. Data sources were obtained from the Web of Science Core Collection only. We only analyzed English studies.

## 5 Conclusion

This study represents the pioneering effort in literature visualization analysis concerning the application of nanoparticles in lung cancer diagnosis using CiteSpace. By conducting a thorough examination of Author/Country/Institutions/Cited Journals/Keyword, we have initially identified and introduced a series of research hotspots and emerging frontiers, including “biomarkers”, “biosensors”, “gold nanoparticles”, and “sliver nanoparticles”. Nevertheless, our analysis was confined to English-language literature within the Web of Science database, which introduces certain limitations. Future investigations in this domain should focus on the synergistic use of various nanoparticles and biosensors to enhance lung cancer detection efficacy and sensor longevity, as well as to delineate the strengths and weaknesses of each material-modified sensor. Moreover, the integrated application of biomaterials and nanomaterials is likely to emerge as a significant research frontier.

## Data Availability

The original contributions presented in the study are included in the article/Supplementary Material. Further inquiries can be directed to the corresponding author.
